# Valence fragmentation dynamics of a promising low global warming etching gas CF_3_CHCF_2_

**DOI:** 10.1038/s41598-025-94119-6

**Published:** 2025-03-19

**Authors:** Tran Trung Nguyen, Toshio Hayashi, Hiroshi Iwayama, Kenji Ishikawa

**Affiliations:** 1 Nagoya University, Furo, Chikusa, Nagoya, 464-8601 Japan; 2 UVSOR Synchrotron Facility, 38 Nishigo-Naka, Myodaiji, 444-8585 Okazaki Japan

**Keywords:** Plasma etching, Selective etching, Hydrofluorocarbon plasma, C_3_HF_5_ (CF_3_CHCF_2_, KSG14), Environmental chemistry, Environmental impact, Applied physics, Chemical physics, Plasma physics

## Abstract

C_3_HF_5_ (CF_3_CHCF_2_, KSG14), a promising low global warming potential (GWP < 1) alternative to traditional perfluorocarbon etching gases for advanced integrated circuit manufacturing, particularly for high-aspect-ratio SiO_2_/SiN stacked layers in 3D flash memory. This study investigates the dissociative photoionization dynamics of C_3_HF_5_ across 10.0–26.0 eV. Ion yield curves and breakdown diagrams reveal that C_3_HF_5_ primarily fragments into C_3_HF_5_^+^, C_3_F_5_^+^, C_3_HF_4_^+^, C_3_F_4_^+^, C_2_F_3_^+^, and CF_3_^+^ ions. Appearance Energies of these fragments, determined from the ion yield curves, indicate fragmentation pathways at low electronic transitions. These findings underscore C_3_HF_5_’s potential as an environmentally friendly etching gas with excellent performance characteristics.

**Supplementary Information:**

The online version contains supplementary material available at 10.1038/s41598-025-94119-6.

## Introduction

The relentless pursuit of miniaturization in semiconductor technology, particularly in 3D flash memory devices, has necessitated the etching of increasingly taller and narrower memory holes^1–3^. This poses significant challenges in achieving precise control over etching processes, including aspect-ratio-dependent etching, selectivity, bowing, distortion, and etch stop^4–8^. To address these challenges, it is crucial to optimize the etching process. This involves controlling ion bombardment energy, ion flux, incident angle, ion species, and etch yields to enhance the etching rates of high-aspect-ratio (HAR) structures^9^. Moreover, managing the complex interplay between reactive radicals, ions, and the polymerized nature of gases is essential for achieving desired etch profiles and material selectivity^10–15^.

The semiconductor industry has been actively seeking alternative gases to replace traditional perfluorocarbons (PFCs), which are potent greenhouse gases with high global warming potentials (GWPs)^16–18^. Fluorinated gases such as hydrofluorocarbons (HFCs) and PFCs such as CF_4_ (GWP = 6500), C_4_F_8_ (GWP = 12400), and CHF_3_ (GWP = 11700)^19^ have been widely used in the semiconductor industry, particularly in etching processes for manufacturing advanced devices such as 3D flash memory. The environmental impacts of etching have driven the search for more sustainable solutions.

C_3_HF_5_ (CF_3_CHCF_2_, KSG14) has emerged as a promising alternative to traditional etching gases owing to its low GWP and high etching performance. With a GWP of less than 1, C_3_HF_5_ demonstrates strong environmental compatibility^20^. Abe et al. demonstrated the effectiveness of C_3_HF_5_ in etching HAR SiO_2_/SiN (ON) layers, achieving enhanced etching rates and precise profile control. Their study compared various C_x_H_y_F_z_ gases, evaluating etch rates, mask selectivity, and practical considerations such as clogging during ON stack reactive-ion etching processes^20^. The impact of dissociated reaction fragments on etched surfaces and the underlying photochemical processes of C_3_HF_5_ remain poorly understood. A detailed understanding of the dissociation pathways and behavior of the reactive species in plasma is crucial for optimizing their use in etching processes^21–26^. Currently, no information is available on the dissociation energy thresholds of fragments from previous studies.

This study employed photoelectron–photoion coincidence (PEPICO) spectroscopy to investigate the dissociative photoionization mechanism of C_3_HF_5_^27–29^. By constructing breakdown diagrams that integrate PEPICO signals for specific ion species, we visualized the fractional abundance of the precursor and fragment ions as a function of photon energy. Analysis of the fragmentation patterns and appearance energies revealed the underlying pathways governing the dissociation of C_3_HF_5_. These insights are crucial for optimizing plasma etching processes in semiconductor manufacturing. By strategically tailoring the gas composition, pressure, and power based on molecular dissociation, desired etch profiles and material selectivity can be achieved. Ultimately, this research paves the way for more sustainable and efficient etching processes by enabling precise control of active species through controlled molecular dissociation and by informing future gas design strategies.

## Experimental setup

PEPICO spectroscopy was used to investigate the photoionization dynamics of C_3_HF_5_. The measurements were conducted at BL3B at the UVSOR facility at the Institute for Molecular Science, Japan. This beamline utilizes a 2.5 m off-plane Eagle-type normal-incidence monochromator, providing tunable vacuum ultraviolet (VUV), ultraviolet, and visible radiation (1.7 to 40 eV). A grating with 1200 lines/mm was used in this study. The beamline combines monochromatization and focusing capabilities, with photon energy resolution serving as a critical parameter for precision measurements. To quantify this resolution, we analyzed the step-like features in the appearance energy spectra of xenon (Xe) near its first ionization threshold (12.130 eV). Ion mass spectra were recorded in 0.001 eV increments across the range of 12.100–12.200 eV using the beamline’s G1 grating, shown in Fig. S1 of Supplementary Information. Three slit configurations—entrance and exit slit widths of 500 μm, 300 μm, and 100 μm—were systematically tested to evaluate resolution dependence on slit geometry. Narrower slit widths produced steeper step edges in the Xe⁺ ion yield, directly correlating with improved energy resolution, shown in Fig. S2 of Supplementary Information. The optimal configuration (100 μm slits) achieved a resolving power (λ/Δλ) of approximately 3000, corresponding to a photon energy resolution of 4.2 meV (full width at half maximum, FWHM), shown in Table S1 of Supplementary Information. Photon Energy resolutions of BL3B evaluated by appearance energy (AE) measurements of Xe^+^ ions shown in the Supplementary Information. The monochromatized VUV radiation was focused using Kirkpatrick-Baez mirrors, bypassing traditional glass capillaries to minimize photon scattering and preserve flux integrity. Beam intensity was monitored in real time using a calibrated AXUV-100 photodiode (IRD Corporation) to ensure stable illumination during measurements. C_3_HF_5_ gas was introduced into the high-vacuum chamber, maintained at a pressure below 3 × 10⁻⁴ Pa, and irradiated with monochromatic VUV light in the energy range from 10 to 26 eV in 0.1 eV increment. Static biasing voltages were applied to the spectrometer plates to extract the photoelectron–ion pairs formed in the interaction region. A three-element lens system focused on the electrons, and the ions were analyzed using a time-of-flight (TOF) mass spectrometer.

**Fig. 1:**
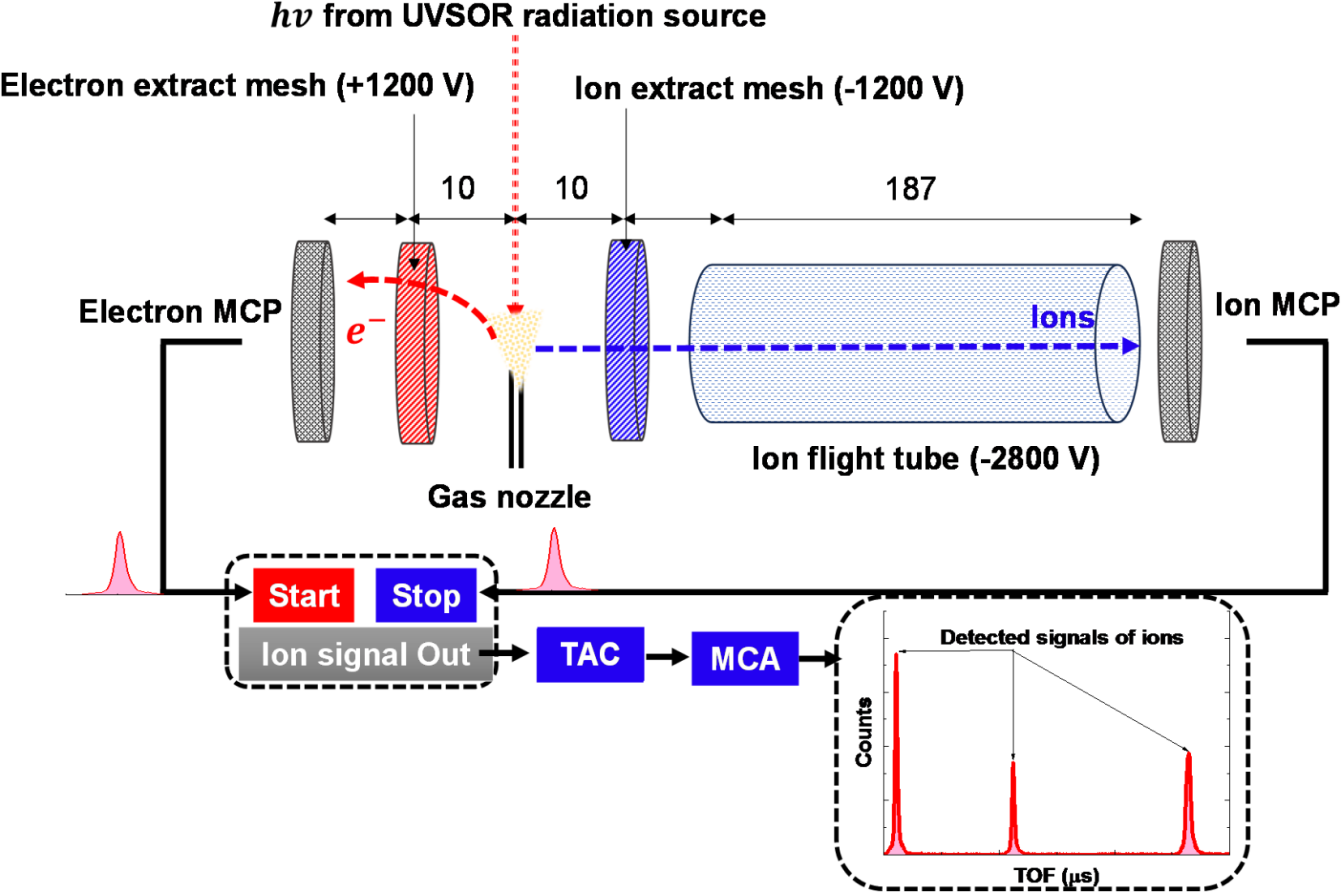
Schematic of the experimental setup for photoelectron–photoion coincidence measurements (MCPs: microchannel plates; MCA: multichannel analyzer; TOF: time-of-flight; dimension unit: mm).

Figure 1 illustrates the TOF spectrometer used in our experiments, optimized for precise ion yield measurements. A gas jet, generated by the capillary nozzle, was irradiated with VUV light between the extraction meshes. The gas source, mounted on an xyz manipulator, enabled adjustments to the source field conditions. The PEPICO technique relies on coincident detection of a photoelectron and its corresponding photoion from the same photoionization event. The detected photoelectrons served as the “start signal” to initiate the TOF measurement for a specific ion. Upon detecting the associated photoions, a “stop signal” was generated. Both electron and ion signals were detected by independent microchannel plate detectors, providing start and stop signals for the TOF spectrum acquisition. A time-to-analog converter (TAC) and a multichannel analyzer (MCA) system determined the TOF for each detected ion. AEs were obtained by extrapolating the ion yield to zero as a function of photon energy. The AEs were determined by performing a linear least-squares fit to the initial rising portion of each ion yield curve. The AE corresponds to the x-intercept of this linear fit, with its uncertainty calculated as the standard error of the x-intercept using Origin’s fitting routine.

To ensure robustness, we also accounted for the photon energy resolution of the beamline, which was maintained at 4.2 meV during the experiment. This resolution-limited uncertainty arises from the intrinsic linewidth of the synchrotron radiation and aligns with the observed tail width of the ion yield curves. The total uncertainty in the AE values was then derived by combining the standard error of the x-intercept and the photon resolution. In our experiments, the effects of higher order harmonics are insignificant, since the energy of the ground electronic state of C_3_HF_5_ lies above that where second-order effects on this beamline are important.

### Typical mass spectra of C_3_HF_5_

**Fig. 2:**
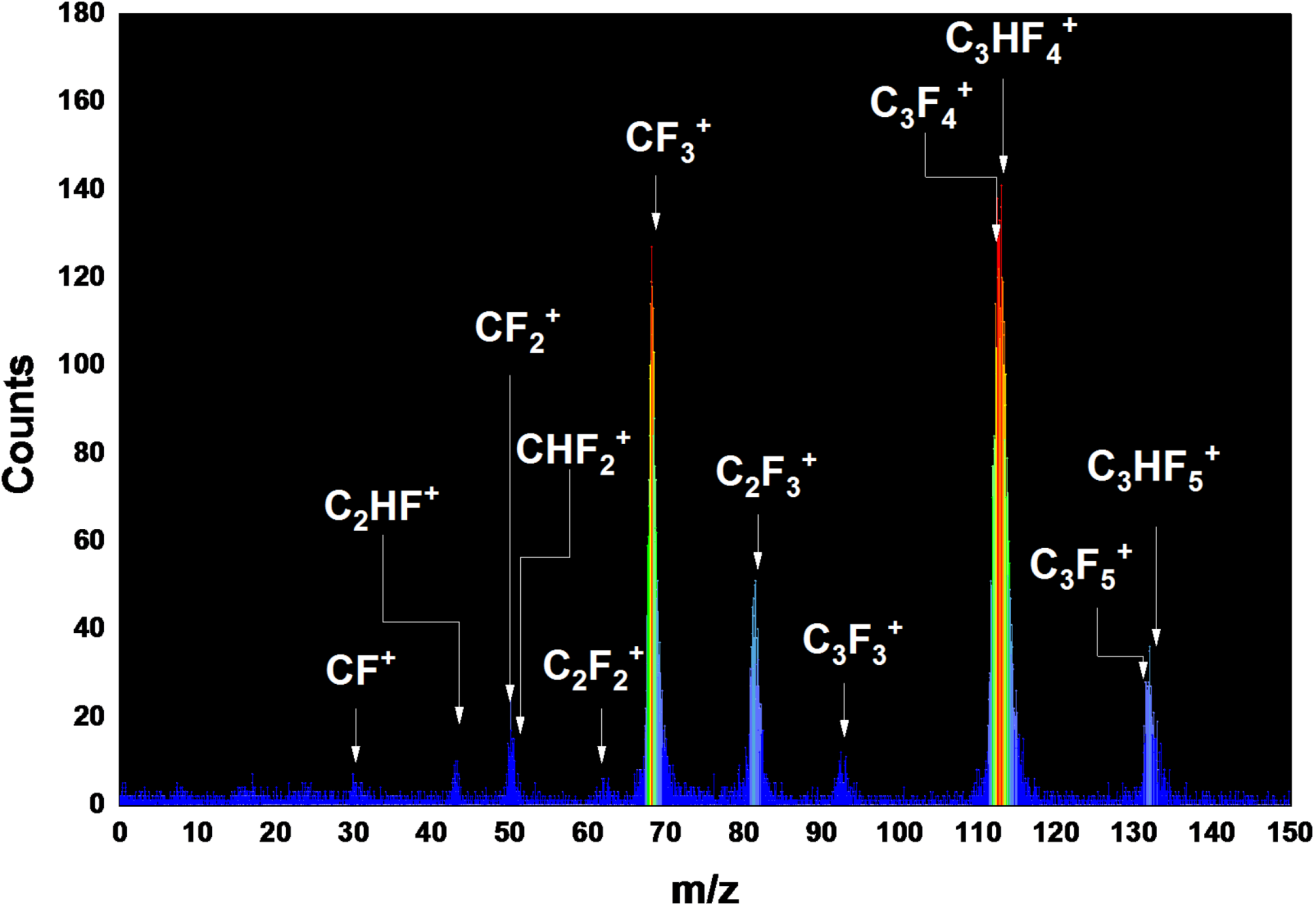
Typical mass spectra obtained from the photoionization of C_3_HF_5_ at photon energies of 20 eV.

Figure 2 shows the positive-ion mass spectrum resulting from the dissociative ionization of C_3_HF_5_ at a photon energy of 20 eV. In particular, the most intense peaks were observed at m/z 113 and 112, corresponding to C_3_HF_4_^+^ and C_3_F_4_^+^ fragment ions, respectively. This suggests that these fragments are the most abundant species in the 20 eV photon excitation mass spectrum. The CF_3_^+^ fragment ion exhibited the third highest intensity, closely following the two preceding ions. The parent ion, C_3_HF_5_^+^, was also detected at a photon energy of 20 eV. Six main fragment ions, namely C_3_HF_4_^+^, C_3_F_4_^+^, CF_3_^+^, C_2_F_3_^+^, C_3_HF_5_^+^, and C_3_F_5_^+^, were identified in the mass spectrum. The fragments, CF^+^, C_2_HF^+^, CF_2_^+^, CHF_2_^+^, C_2_F_2_^+^, and C_3_F_3_^+^ appeared weakly at a photon energy of 20 eV.

### Ion yield curves and breakdown diagram for photoionisation of C_3_HF_5_

**Fig. 3:**
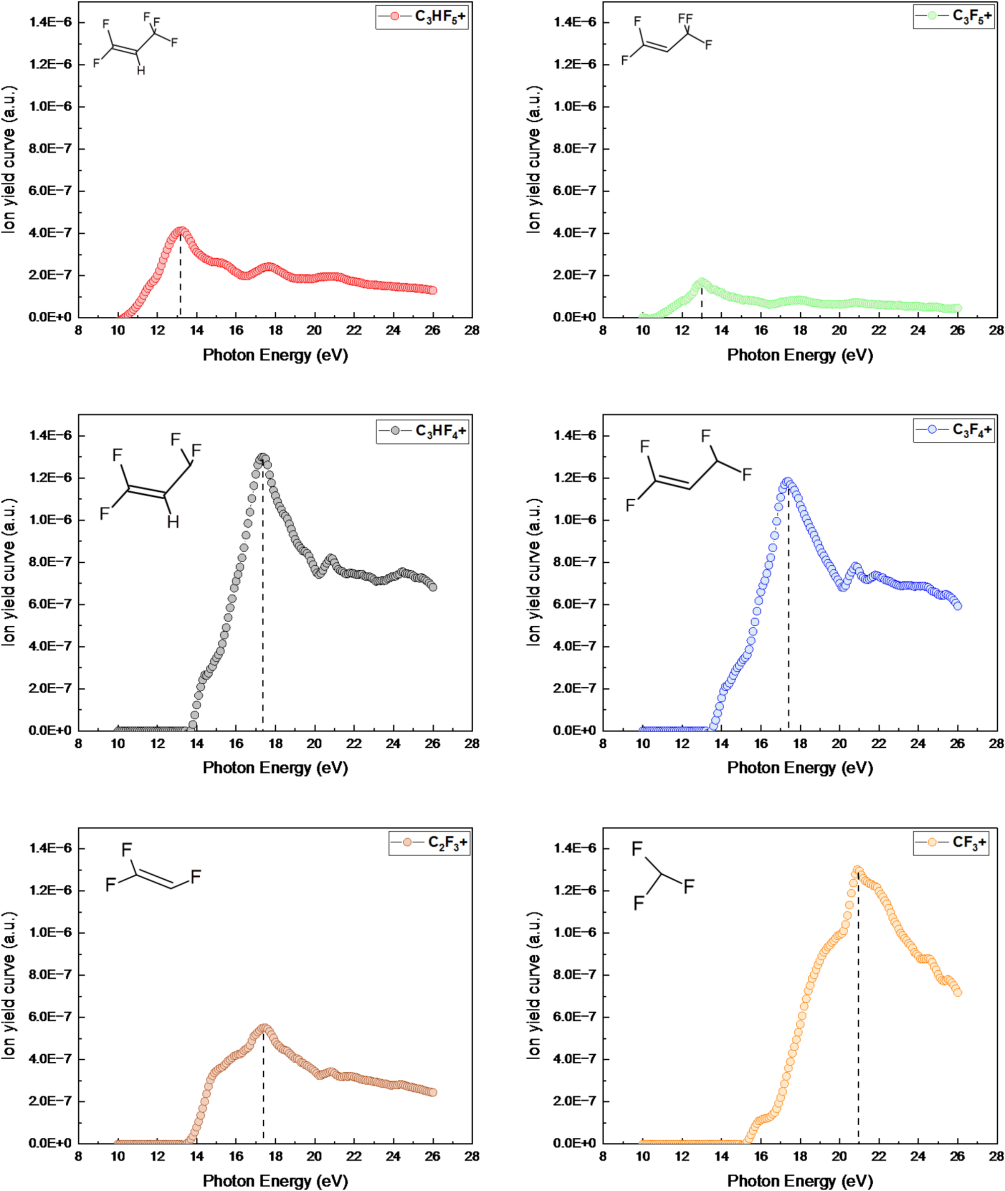
Ion yield curves of the fragment ions produced from the photoionization of C_3_HF_5_. A photon energy resolution of 4.2 meV.

**Fig. 4:**
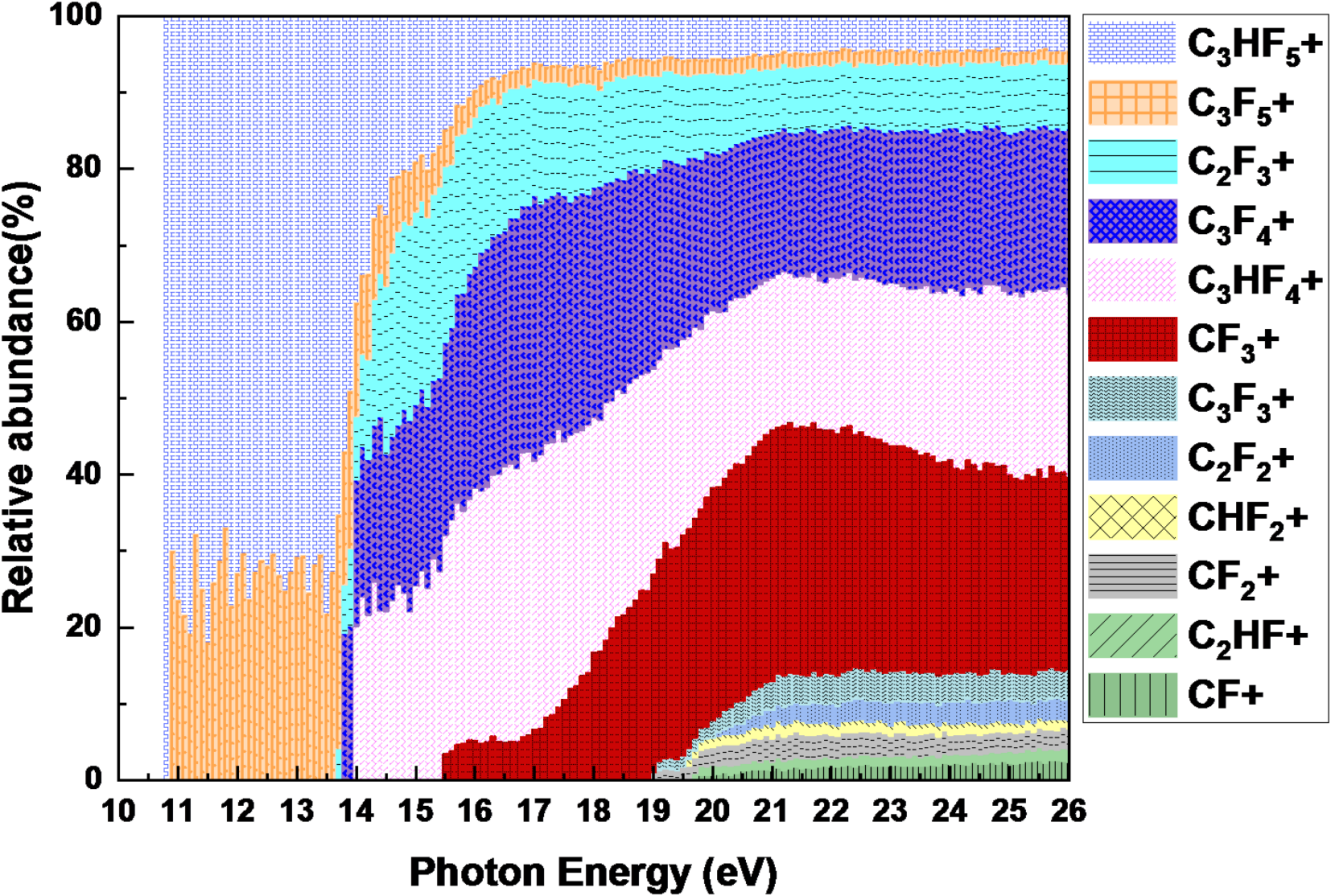
Relative abundance curves of the fragment ions produced from the photoionization of C_3_HF_5_. A photon energy resolution of 4.2 meV.

A scanning energy PEPICO spectrum of C_3_HF_5_ was acquired over a photon energy range of approximately 10.0 to 26 eV, using a constant step size of 0.1 nm. The optimal configuration (100 μm slits) achieved a photon energy resolution of 4.2 meV. Figure 3 shows the ion yield of C_3_HF_5_ as a function of the photon energy, spanning from 10 to 26 eV, normalized to the photon flux of the VUV light source. Figure 4 shows a breakdown diagram constructed from the ion yield curves, illustrating the relative ion abundance as a function of the photon energy resulting from the dissociative ionization of C_3_HF_5_.

At photon energies below 14 eV, the parent ion, C_3_HF_5_^+^, dominated the ion yield, accounting for approximately 80% of the detected ions. The remaining fragment ion, C_3_F_5_^+^, constituted approximately 20% of the ion yield before the other fragments became significant. A significant shift in the fragmentation pattern was observed as the photon energy increased from 14 to 15.5 eV. The fragment ions C_2_F_3_^+^, C_3_HF_4_^+^, and C_3_F_4_^+^ became the dominant species, contributing to approximately 20%, 20%, and 14% of the total ion yield, respectively. Concurrently, the abundances of the parent ion C_3_HF_5_^+^ and fragment ion C_3_F_5_^+^ decreased dramatically, with average yields of approximately 15% and 3%, respectively. A significant increase in the abundance of CF_3_^+^ fragment ions was observed at higher photon energies, with the most pronounced contribution occurring between 19 and 26 eV. The CF_3_^+^ ion accounted for an average of approximately 25% of the total ion yield within this energy range. More small fragments, likely resulting from secondary fragmentation pathways, were first detected at 20 eV; however, their contributions to the overall ion yield were minimal, averaging only 2–3%.

The main dissociation fragments dominated the ion yield, accounting for over 85% of the total ion population. Secondary fragments from more complex dissociation pathways emerged at higher photon energies and contributed only a minor fraction to the overall ion yield. The photoionization cross section of dissociated fragments, combined with simulations, plays a crucial role in understanding and optimizing the etching processes in semiconductor manufacturing. These data provide valuable insights into the fundamental physics and chemistry of plasma–surface interactions.

### Fragment ion appearance energies of C_3_HF_5_

**Fig. 5:**
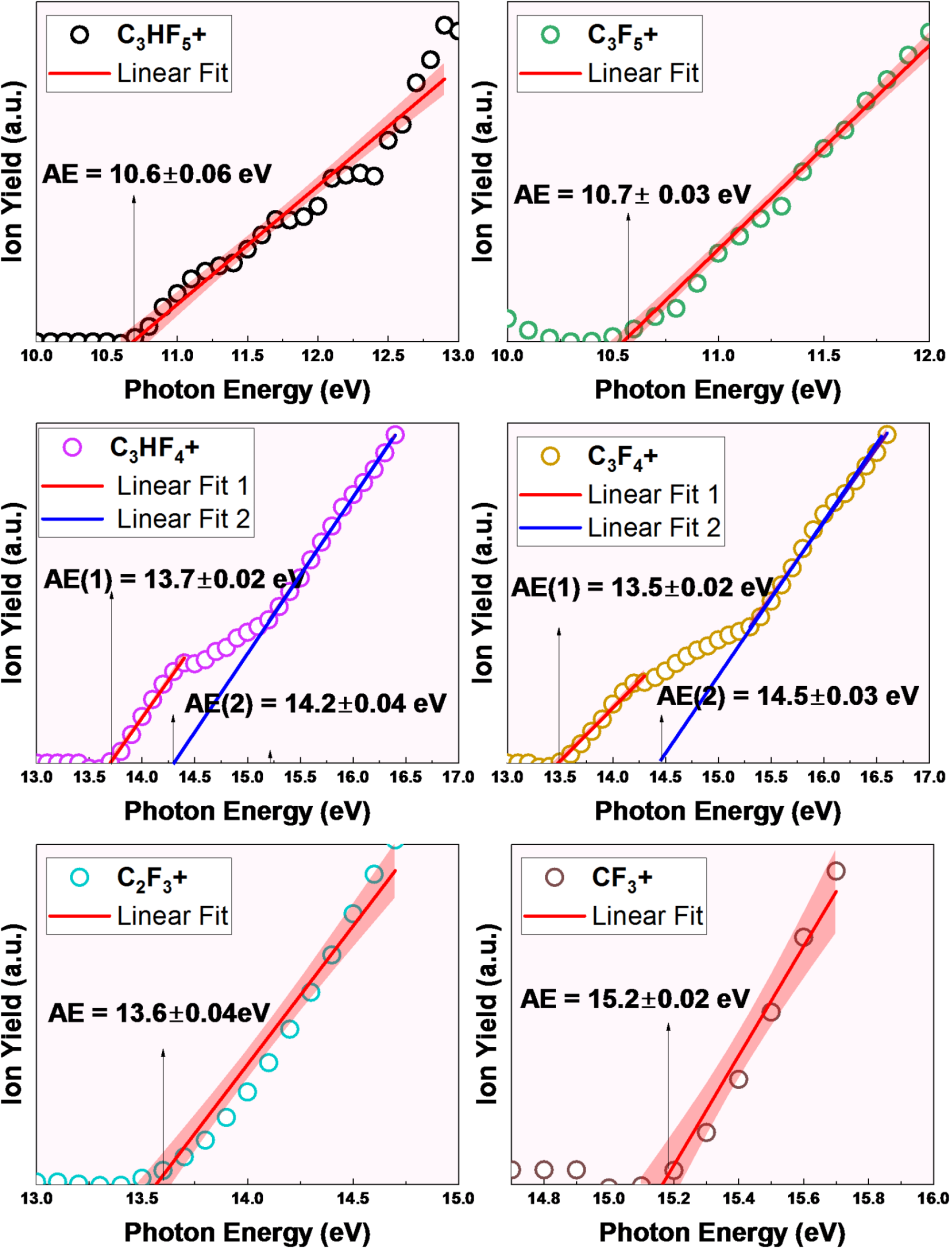
Threshold photoion yield curves for main fragment ions produced from C_3_HF_5_.

Figure 5 shows the threshold photoion yield curves corresponding to the main fragment ions. Table 1 presents a comprehensive list of the significant fragments identified within the investigated photon energy range, including their corresponding appearance energies as measured in this study. The AE of each fragment ion was determined by extrapolating the linear region of the ion yield curve to baseline. Given the experimental resolution, this corresponded to the initial onset of the ion signal. The results show that the parent ion, C_3_HF_5_^+^, appeared at the lowest energy with an AE of 10.6$\:\pm\:$0.06 eV, which suggested a direct photoionization process: C_3_HF_5_ + hv → C_3_HF_5_^+^ + e−. At a slightly higher energy of 10.7$\:\pm\:$0.03 eV, the ion C_3_F_5_^+^ was observed, corresponding to H loss, C_3_HF_5_ + hv → C_3_F_5_^+^ + H+ e−. This H$\:-$ loss originated from the CH$\:-$ group. This fragmentation process appeared to be straightforward, involving C–H bond cleavage without significant rearrangement of the carbon atoms. This process is energetically favorable. The ion yields of both ions peaked at a photon energy of 13 eV and subsequently decreased with increasing photon energy.

Subsequently, fragments C_3_HF_4_^+^ (F loss), C_3_F_4_^+^ (HF loss), and C_2_F_3_^+^ (CHF_2_ loss) appeared at approximately 14 eV. Notably, the curves for C_3_HF_4_^+^ and C_3_F_4_^+^ exhibit distinct changes in slope, indicating the opening of new fragmentation channels at higher photon energy. This observation can be explained by the presence of multiple fragmentation pathways. For instance, the C_3_HF_5_ molecule (CF_3_-CH-CF_2_) can dissociate via two distinct routes, namely CF_2_-CH-CF_2_ and CF_3_-CH-CF, both yielding C_3_HF_4_⁺. These pathways possess different activation energies due to the varying C-F bond strengths in the CF_3_ and CF_2_ groups. Consequently, two distinct AEs are observed for C_3_HF_4_^+^: 13.7$\:\pm\:$ 0.02 eV and 14.2$\:\pm\:$0.04 eV, corresponding to the respective onset of these pathways. Similarly, C₃F₄⁺ displays a change in slope due to analogous fragmentation routes, with AEs determined to be 13.5$\:\pm\:$0.02 eV and 14.5$\:\pm\:$0.03 eV.

The change in slope observed for the parent ion (C_3_HF_5_^+^) at higher photon energies, while less straightforward, may be attributed to the population of a different electronic state of the parent ion or a change in the Franck-Condon overlap. This could lead to a change in the ionization cross-section, resulting in the observed inflection point in the ion yield curve. C_2_F_3_^+^ ion (AE = 13.6$\:\pm\:$0.04 eV) became increasingly prominent as the photon energy increased. The loss of F from C_3_HF_5_^+^ was the most intense primary dissociation channel, accounting for approximately 45% of the fragmentation at its peak. This suggested that the dominant photodissociation pathway was C_3_HF_5_ + hv → C_3_HF_4_^+^ + F + e−. This indicated a direct ionization origin for C_3_HF_4_^+^. The yield of the C_3_HF_4_^+^ ions peaked at an energy of 17$\:\pm\:$0.05 eV. CF_3_^+^ appeared slightly above 15.2$\:\pm\:$0.02 eV and became the predominant ion at energies exceeding 19 eV. This suggests that CF_3_^+^ was the primary dissociation channel. Other fragment ions, including CF^+^, C_2_HF^+^, CF_2_^+^, CHF_2_^+^, C_2_F_2_^+^, and C_3_F_3_^+^, were observed at photon energies of approximately 20 eV, with relatively low intensities. These fragments appeared to be the primary products of secondary dissociation processes, resulting from more complex fragmentation pathways involving multiple bond cleavages and rearrangements. Further investigation is required to elucidate their precise formation mechanism.

Plasma processing typically uses electron energies of 1 ~ 5 eV. To maximize the yield of target ions, such as C_3_HF_4_^+^, C_3_F_4_^+^, CF_3_^+^, C_2_F_3_^+^, and C_3_F_5_^+^, we focused on photoionization processes using lower photon energies, closer to the ionization threshold. These fragments were considered the primary dissociation fragments of C_3_HF_5_^+^, as they were directly formed from the parent ion.

**Table 1:** List of the significant ion fragments and their appearance energies.

$\:\varvec{m}/\varvec{z}$	Ionic fragment	Appearance energy (eV)
132	C3HF5^+^	10.6 ± 0.06
131	C3F5^+^	10.7 ± 0.03
113	C3HF4^+^	13.7 ± 0.02 14.2 ± 0.04
112	C3F4^+^	13.5 ± 0.02 14.5 ± 0.03
69	CF3^+^	15.2 ± 0.02
81	C2F3^+^	13.6 ± 0.04
93	C3F3^+^	17.9 ± 0.1
62	C2F2^+^	20.0 ± 0.1
51	CHF2^+^	19.5 ± 0.1
50	CF2^+^	17.9 ± 0.1
44	C2HF^+^	19.5 ± 0.1
31	CF^+^	19.5 ± 0.1

## Discussion

The etching of HAR structures, particularly for 3D NAND flash memory devices, requires precise control over etching processes to ensure high selectivity and accurate feature definition. C_3_HF_5_ gas has emerged as a promising alternative to traditional PFC gases owing to its low global warming potential. The dissociation of C–H and C–F bonds in HFC molecules plays a crucial role in determining the etching and deposition behaviors. Fluorine (F) atoms generated by C–F bond dissociation are highly reactive and can etch silicon (Si) surfaces. Hydrogen (H) atoms produced by C–H bond dissociation can promote polymer deposition on the substrate surface, which can enhance selectivity and improve process accuracy. Additionally, the C_x_F_y_ and C_x_H_y_F_z_ radicals formed from C–F and C–H bond dissociation can contribute to polymer deposition, particularly those formed via F abstraction. To achieve an optimal etching performance, a delicate balance between the etching and deposition processes is necessary. This balance is influenced by the relative concentrations of the reactive species, including F atoms, H atoms, C_x_F_y_ and C_x_H_y_F_z_ radicals. The dissociative photoionization of C_3_HF_5_ provides valuable insights into its fragmentation pathways and the potential for generating reactive species suitable for plasma etching processes.

**Fig. 6:**
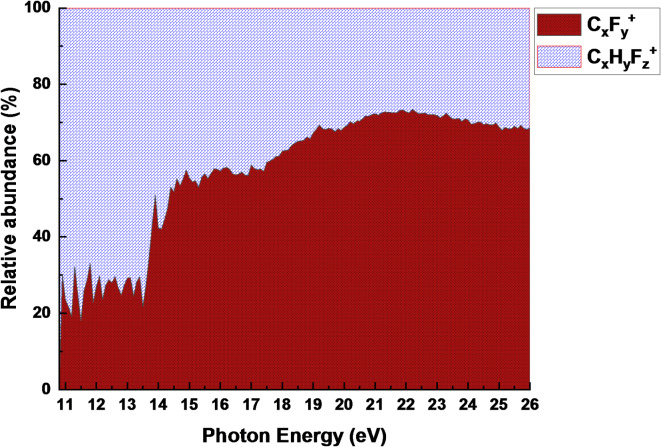
The relative abundances of C_x_F_y_^+^ and C_x_H_y_F_z_^+^ ions produced from C_3_HF_5_ as a function of photon energy.

Figure 6 illustrates the relative abundances of C_x_F_y_^+^ and C_x_H_y_F_z_^+^ ions as a function of photon energy. At photon energies between 10 and 14.5 eV, C_x_H_y_F_z_^+^ ions dominated the ion spectrum at comprising approximately 70%, while C_x_F_y_^+^ ions accounted for the remaining 30%. Notably, these results aligned well with the relative abundances of C_x_H_y_F_z_^+^ and C_x_F_y_^+^ ions detected by quadrupole mass spectrometer in plasma environments^20^. The formation of C_3_HF_4_^+^ was attributed to the direct C–F bond cleavage. A smaller fraction of C_3_F_5_^+^ was also observed, which resulted from direct C–H bond scission. Primary dissociation fragments such as C_3_HF_4_^+^, C_3_F_4_^+^, CF_3_^+^, C_3_HF_5_^+^, and C_3_F_5_^+^ are considered to play crucial roles in the etching of SiO_2_ and SiN films. When the photon energy exceeded 20 eV, C_x_F_y_^+^ ions such as CF_3_^+^ and smaller fragments became increasingly abundant. These ions likely originate from complex fragmentation pathways involving multiple bond cleavages and rearrangements. Although their reactivity toward etching may be lower, they can still contribute to the overall etching process and influence the formation of radicals and other reactive species.

**Fig. 7:**
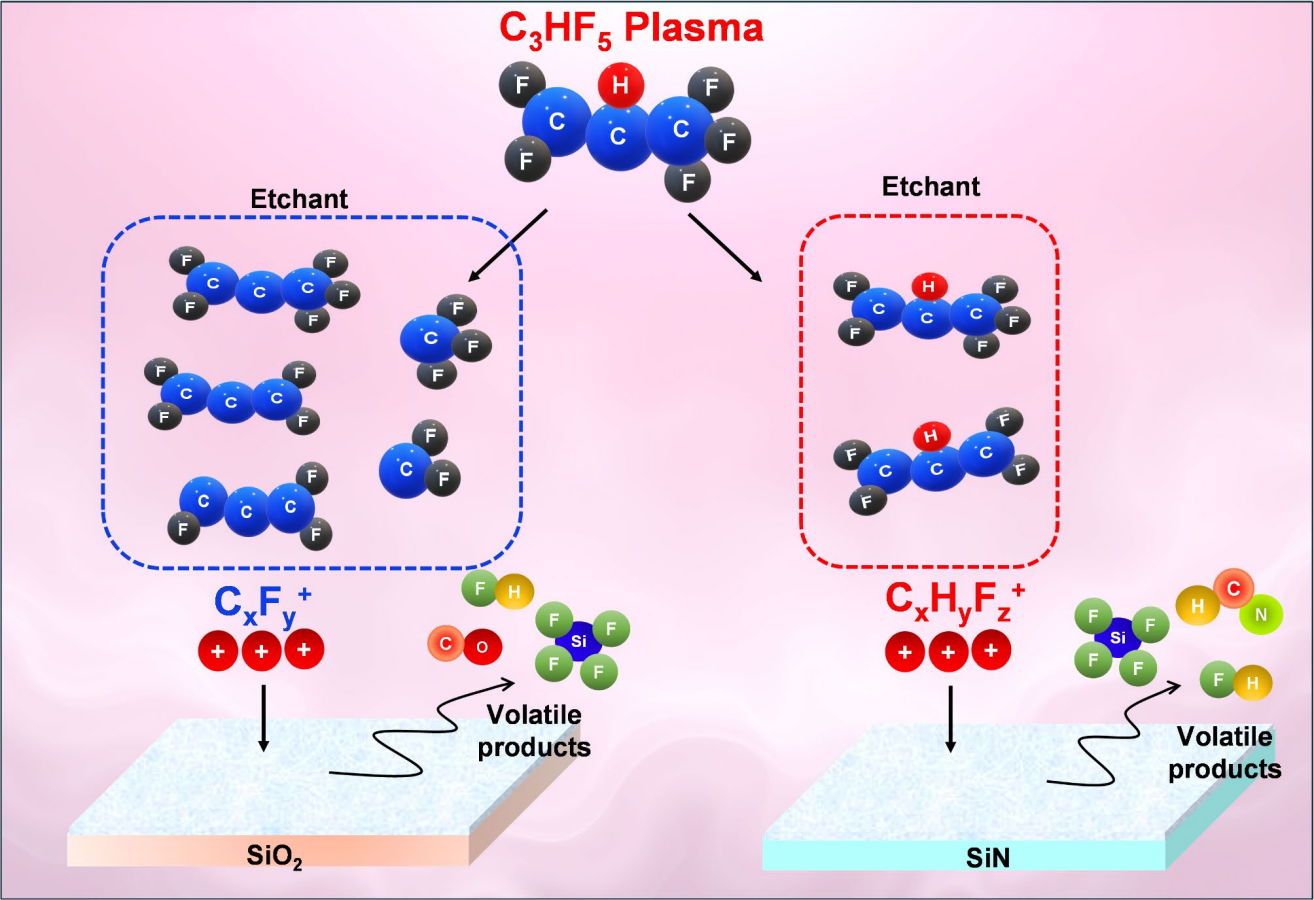
Model for etching SiO_2_ and SiN films in C_3_HF_5_ plasma chemistry.

Figure 7 proposes a model for the mechanism of ion and radical generation in the C_3_HF_5_ plasma. C_3_HF_5_ provides a rich source of reactive species C_x_H_y_F_z_^+^ ions, with forms such as C_3_HF_5_^+^ and C_3_HF_4_^+^, an effective stoichiometric balance between H and F for etching SiN layers, and C_x_F_y_^+^ ions, with forms such as C_3_F_4_^+^ and CF_3_^+^, for etching SiO_2_ layers in HAR plasma etching. By comprehending the fragmentation patterns and relative abundances of different ion species, we can optimize the plasma etching process by tailoring the photon energy to generate the desired ion species and achieve specific etch profiles and selectivities.

## Conclusion

The dissociative ionization of C_3_HF_5_ was examined using the PEPICO technique in the energy range of 10–26 eV. The main fragment ions observed were C_3_HF_4_⁺, C_3_F_4_⁺, CF_3_⁺, C_2_F_3_⁺, C_3_HF_5_⁺, and C_3_F_5_⁺. The AEs of these ions were determined from their ion yield curves. At lower photon energies (10–14 eV), C_3_HF_5_⁺ was the most abundant ion, generated through dissociative ionization with a threshold energy of approximately 10.7 eV. At higher energies (up to 18 eV), C_3_HF_4_⁺ became dominant. Beyond 18 eV, CF_3_⁺ ions became increasingly abundant. The findings of this study provide valuable insights into the dissociation pathways of C_3_HF_5_ and their potential roles in the etching of SiN and SiO_2_ films. A deeper understanding of these fragmentation processes can aid in optimizing the selectivity control in plasma etching processes, leading to improved HAR etching performance. This information may also be useful for controlling the generation of fluorocarbon ions and designing novel HFC molecules.

## Electronic supplementary material

Below is the link to the electronic supplementary material.

Supplementary Material 1

## Data Availability

The raw data supporting the findings of this study are available from the corresponding author upon reasonable request.
