# Supplementary material for: Valence fragmentation dynamics of a promising low global warming etching gas CF3CHCF2
Source: Sci Rep. 2025 Mar 19;15:9507. doi: 10.1038/s41598-025-94119-6 (PMC11923280; doi:10.1038/s41598-025-94119-6)
Supplement: Supplementary file 1 — Supplementary Material 1 [file 41598_2025_94119_MOESM1_ESM.pdf]

Supplementary Information for

Valence Fragmentation Dynamics of a Promising Low Global Warming Etching Gas CF_3CHCF_2

Tran Trung Nguyen[1]*, Toshio Hayashi[1], Hiroshi Iwayama[2], and Kenji Ishikawa[1†]

[1] *Nagoya University, Furo, Chikusa, Nagoya 464-8601, Japan*

[2] *UVSOR Synchrotron Facility, 38 Nishigo-Naka, Myodaiji, Okazaki, 444-8585, Japan*

**Corresponding Author E-mail address: tran.trung.nguyen.y7@f.mail.nagoya-u.ac.jp*

[†] *E-mail address: ishikawa@plasma.engg.nagoya-u.ac.jp*

List of Tables

Table S1. Photon energy resolutions for G1 grating……………………………………………………………3

List of Figures

Figure S1. Typical time-of-flight mass spectrum of Xe ion……………………………………………………3

Figure S2. Xe ion yield spectra around the ionization energy of 12.130 eV for three slit configurations……..3

Photon Energy resolutions of BL3B evaluated by appearance energy measurements of Xe^+ ions

A beamline in synchrotron facility generally works as a monochromator and focus system. A photon energy resolution of the beamline is one of the most important parameters for various measurements. To maintain the performance of the beamlines, it is desirable to check the actual photon energy resolution regularly.

The beamline BL3B can provide monochromatic light in the range from visible to extreme ultraviolet region. Previously the photon energy resolution of BL3B was evaluated by vibration-rotation spectra of oxygen molecules [1], which are significantly complex. In this work, we estimate photon energy resolutions of BL3B from appearance energy spectra, which have step functional structure at an ionization threshold.

The photoelectron-photoion coincidence (PEPICO) measurements for an ion time-of-flight mass spectrum were performed at UVSOR BL3B. A schematic view of PEPICO measurements is described in the previous report [2]. Sample gas is xenon, whose ionization threshold is 12.130 eV [3]. The ion mass spectra were recorded at photon energies ranging from 12.100 eV to 12.200 eV in 0.001 eV-increments. We used G1 grating of BL3B. We estimate photon energy resolutions for three slit configurations of (entrance slit, exit slit) = (500μm, 500μm), (300μm, 300μm) and (100μm, 100μm).

Figure S1 shows a typical mass spectrum of Xe ions. The peak structure results from an isotope distribution of Xe atoms. The corresponding mass resolution m/Δm is 400.

Figure S2 shows Xe ions yield spectra for three slit configurations. We find steep rises at the ionization threshold of 12.130 eV. With narrowing slit sizes, steps of Xe ion yield spectra become steeper. This shows that photon energy resolution become better. From three spectra, we estimate photon energy resolutions for three slit configurations and summarize Table S1. In future, we also perform estimations of photon energy resolutions for G2 and G3 gratings.

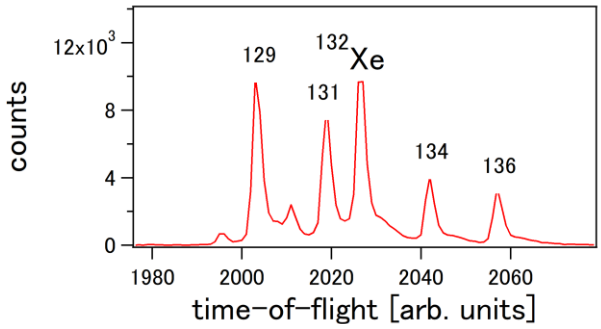

Fig. S1. Typical time-of-flight mass spectrum of Xe ions.

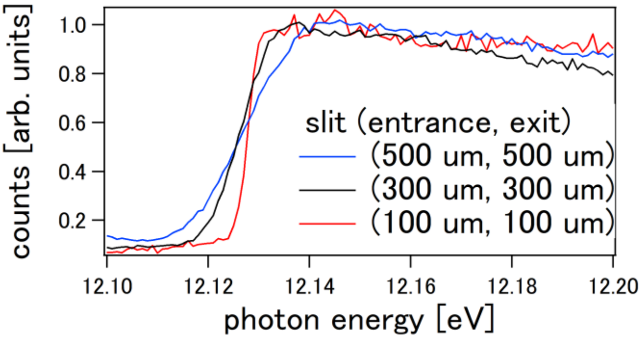

Fig. S2 Xe ion yield spectra around the ionization energy of 12.130 eV for three slit configurations.

Table S1. Photon energy resolutions for G1 grating

Slit size	ΔE @ hν=12eV	E / ΔE
(500um, 500um)	20 meV	600
(300um, 300um)	11 meV	1000
(100um, 100um)	4.2 meV	3000

[1] K. Fukui, et al., J. Synchrotron Rad. **21** (2014) 452.
[2] H. Iwayama and T. Horigome, UVSOR Activity Report **48** (2021) 41.
[3]https://physics.nist.gov/PhysRefData/ASD/levels_form.html.
